# Social Hysteria

**Published:** 1908-05

**Authors:** G. Frank Lydston

**Affiliations:** Professor of Genito-Urinary Surgery, State University of Illinois; Professor of Criminal Anthropology, Chicago Kent College of Law


### TEXAS MEDICAL JOURNAL.

**Established July, 1885.**

F. E. DANIEL, M. D., - - - - Editor, Publisher and Proprietor

PUBLISHED MONTHLY.—SUBSCRIPTION $1.00 A YEAR.

VOL. XXIII. AUSTIN, MAY, 1908. No. 11.

The publisher is not responsible for the views of contributors.

For Texas Medical Journal.

### Social Hysteria.

BY G. FRANK LYDSTON, M. D.,

Professor of Genito-Urinary Surgery, State University of Illinois; Professor of Criminal Anthropology, Chicago Kent College of Law.

To the student of sociology the present condition of social unrest in the United States is very interesting. To the casual observer, the momentous events of the past few years appear as isolated phenomena. The "bull in the China shop" explosions of Theodore Roosevelt, the meteoric flight of Judge Landis, of Chicago, from obscurity into world prominence, the anti-corporation legislation in general and the anti-railroad legislation in particular, the present wave of political reform and anti-graft agitation, the widespread prohibition movement, the Averbuch-Shippy tragedy in Chicago and the bomb explosion in Union Square, New York, have been generally accepted as the result of individual action rather than as manifestations of social unrest.

Our worthy President takes himself and is taken by the world too seriously. Grumblings of protest by the underdog were loud in the land while he was but a puling child in arms—even before he was born. Roosevelt, the man, arrived on the scene when the American social atmosphere had become surcharged with protest and was used very much as was Franklin's key and kite, to draw the lightning from the lowering social clouds. In the long ago Grant saw the menace to our country hidden in "vested interests," but even had he possessed Roosevelt's energy and lack of balance he would have been impotent to change the conditions against which he protested. The time was not yet ripe.

Had Roosevelt appeared on the scene, even ten years ago, and undertaken his present strenuous rôle of regulator of the universe, he would have been regarded as a more dangerous man than the reddest of the "Reds." When the time was ripe, the seeds of hysterical resentment against wrong thoroughly sown and the nail set, the country—unconsciously as to both parties to the act—used Roosevelt as the hammer to drive it home. Protest of the "outs" against the "ins" had finally crystallized, had become dynamized and focalized itself in Roosevelt. The reform wave once started spread in all directions and now threatens to engulf everything from the insurance swindler to the keeper of the doggery.

Social reforms move in waves and come in epochs. Mob hysteria was behind the Reformation in England and the French Revolution; it lies behind the present prohibition movement and lawmaking craze in America alike. Like most manifestations of mob dynamics, it is open to suspicion as to its intelligence and logicality. Mass reason is merely mass impulse.

It is a far cry from Roosevelt to the arch Red, but their psychology is identical. Both are leaders of the "mob"—crowd heroes.

Roosevelt's psychology in an illiterate, downtrodden underdog might produce an anarchist—a man in whom protest against the social conditions of the underdog crystallizes—focalizes in acts of violence against the higher and more powerful social sphere that overlies and crushes him down. Such a man takes no account of his own inherent unfitness in life's battle.

Poor crazy Averbuch was an illogical, murderous instrument of protest of the proletariat against the powers that govern for the "powers that prey." The hereditary impressions and rebellious instincts which centuries of past and years of personal racial and social oppression and outrage had engendered in him were so thoroughly ingrained in him that anything savoring of authority was to his cracked brain a thing to be destroyed, even at the price of self-destruction. His attack on Chief Shippy was natural, even if illogical.

No well-balanced individual ever believed that by a single murderous act of his own or by self-immolation he could right the wrongs of his race or social plane. The "martyrs" of history were much alike in their psychology, and many of them were in nowise different from the vulgar assassins of both ancient and modern times. Fanaticism underlay the acts of practically all of them.

Jeanne D'Arc and Charlotte Corday, Marat, Danton, Robespierre, John Wilkes Booth, Guiteau, Czolgoz, the assassins of King Carlos,

Averbuch and Silverstein were of the same psychic kidney. Their psychology multiplied in the form of a crowd has always stood, and will always stand, for revolution and social "reform"—primarily for social unrest and disorder. From social unrest to social hysteria is but a step.

Rooseveltism in ages past burned and was burned at the stake, organized inquisitions and St. Bartholomew-fests, stabbed "tyrants"—and by the same token was sometimes on the other end of the knife in that grand old drama, *sic semper tyrannis.* It requires no great flight of the imagination to conceive of our idol, Theodore, in full panoply of war at the head of a crusa[illegible], swearing upon his cross-handled sword the destruction of the hated moslem enemies of the true cross. He once shot a fleeing Spaniard and bragged about it, which, let us hope, was evidence of mental excitement rather than of medieval bloodthirst. If one could but forget his exalted station, fancy might picture him astride his faithful Rosinante charging furiously on windmills. Might one imagine Taft as Sancho Panza by conjuring up an elephant in lieu of that faithful donkey? Whatever rule he might essay, Roosevelt with the hysterical crowd behind him—the epoch permitting—would present a far different picture than that which the nation now sees in its morning papers. And let it not be thought that I altogether disapprove of him or his policies. I admire him greatly, but "a cat may look at a king," and perchance comment upon him, and, besides, there is such a thing as an overdose of social alteratives or eliminants.

To return to Averbuch and the statement I have made that his attack on Chief Shippy was a natural result of his psychology. What was the inevitable result on the minds of such crack-brains as Averbuch of the part played by the police in the Reitman parade? To their ill-balanced intellects the search of the American eagle as they had heard it in Europe promised something. It promised freedom of speech and assemblage. Some of us are wiser in our interpretation of the Constitution and can read between the lines. With the militarism and intolerence of Europe fresh in mind the misguided anarchistic and socialistic fools saw in the police interference a repetition of the slaughter of the innocents perpetrated by the minions of the Czar on the occasion of the procession of harmless and impotent protest headed by Father Gapon, the priest and leader whom they idolized. The attempt on the life of Chief Shippy was a logical sequence.

What I have said of Averbuch equally applies to Silverstein, the luckless bomb thrower of Union Square. Underlying the mad acts of such would-be regulators of social conditions is social hysteria. This also underlies the excitement and resentment caused by such attempted murders. In minor degree the recent hysterical social resentment against anarchy was a repetition of that of the long ago which sent the Chicago anarchists to the gallows. Its crystallization into a morbid fear of the tongue of the queen of anarchistic gab-feasts, Emma Goldman, was a spectacle for gods and men.

The taking of addle-pated Reitman as seriously as he takes himself was a huge joke, worthy of the pen of a Moliere. The club as a moral persuader for the anarchist in action is a potent remedy, the muzzle for his oratory never. Let the degenerates shriek. The escape of neuropathic steam relieves such part of social hysteria as in them lies. But before either muzzle or club, should come food and raiment, and opportunity to work—even "work compulsion."

The waning of the power of established religion has had much to do with social unrest and hysteria. In his struggle toward the ideal, man takes varied and devious paths. The old forms of religion are no longer satisfying. People think more and follow less—form their own attitude of mind and do not so readily don shop-worn mental garb—hence ancient creeds are no longer popular. Man sees the light and is struggling toward a betterment of his condition here on earth, struggling for the attainment of his ideals in the present. He is not so concerned as he once was with the hereafter. His ideas of betterment are often fallacious, even pernicious, it is true, and he is not seldom prone to take the wrong road, but the mainspring of his actions is the same.

Christian Science, Dowieism, Socialism, anarchy in all forms and many other more or less tolerated cults are rooted in the depths of the same psychic conditions. Full-bellied Krapotkin preaches anarchy by evolution, Herr Most and Emma Goldman are the apostles of anarchy by revolution, fire and blood. Both types are conscientious enough, each according to his lights. There is an inherent difference of brain structure behind the difference in method. Perhaps the difference in method is largely a matter of immediate brain nutrition.

The psychology of the well-fed, well-clothed and well-housed anarchist may well be philosophic. He can afford to wait. He has no hungry belly to sound the charge on society. Not so your Averbuchs and Czolgozes. They are flat-bellied, lean and hungry kine and prone to break into the other fellow's clover patch as best they may and at whatever cost of fire and blood.

As I have already stated, in effect, there is a question in my own mind as to whether a policy of repression of gab-fest social protest is wise. England is the haven of refuge for the world's anarchists. She holds them responsible for their acts, not for their words, and today enjoys an immunity from anarchistic outrage such as few other countries enjoy.

What ruler is so safe as is King Edward? Ours? Let the shades of Lincoln, Garfield and McKinley answer. It is noteworthy that the Haymarket massacre, the Averbuch tragedy and the recent explosion of the bomb in Union Square, New York, were the direct outcome of police attempts to suppress anarchy by interfering with things which in themselves were harmless and in violation of no law—things in which misguided fools who take the Constitution of the United States literally were merely exercising their prerogatives as American citizens. Had the anarchist processions and gatherings been riots by trades unionists, the police would probably have stood idly by and listlessly watched, quite as a matter of course, the slugging of inoffensive citizens.

There are certain phases of social hysteria and unrest that are appalling to the patriot who believes that America is a land of justice, freedom and equal rights. During the teamsters' strike in Chicago I witnessed from the sidewalk the funeral of American liberty—goods vans solemnly escorted through the streets of Chicago by police. Then and there I thought of the tea party of old time in Boston harbor, and murmured *"cui bono?"*

Our social system is mildly tolerant of the hysteria of organized labor—because it is afraid of it. It is intolerant of anarchy because it is also afraid of it. The difference in treatment of the two evils is illustrated by the lonely unarmed man who meets a hungry tiger and a venomous snake in the wilderness. He is afraid of both, but he hides or runs away from the one and raps the other on the head with a stick. He is bigger than the snake, but—

When hysteria attacks the upperdog with sufficient violence, he may rush like an avalanche on the hysterical social tiger. He may or he may not overestimate his own strength—he may or may not destroy himself. Time will tell. Meanwhile let him reflect that the aggregate loss of life and property incidental to great industrial strikes in America has been a thousandfold greater than that of anarchy.

Did Emma Goldman in her most frenzied moments ever perpetrate anything so pregnant with murderous and destructive possibilities as this:

"Every union should have a rifle club. I strongly advise you to provide every member with the latest improved rifle, which can be obtained from the factory at a nominal price. I entreat you to take action on this important question, so that in two years we can hear the inspiring music of the martial tread of 25,000 armed men in the ranks of labor."

This from a public speech by the President of the Western Federation of Miners! As to its results, read its bloody record.

It is the writer's belief that the stronger cults which are today agitating society and pervading it with hysteria must have their day. Socialism is the religion of the future, and must have its "try out." Thinkers, paranoiacs and degenerates of all kinds, mental clods, down-and-outs, heretics who have drifted away from established faiths, wage-earners, philanthropists, and demagogues—in short, everybody whose selfish interests are not already well conserved, will flock to its standard, and the country will be submerged in a hysterical wave of change and imbecile reform which the "full-bellies" can not stem. Upon the crest of the wave will ride a "popular idol"—his destination the White House. Should he chance to be a demagogue—which is likely—heaven help us.

But, all the same, the cult must have its "try out"—the socialistic dog must have his day—and he will have it when social neurosis has culminated in a hysterical storm of sufficient power. As to whether the country will be better or worse for the experiment—*quien sabe?*

Possibly Roosevelt has had his "ear to the ground," and is riding on the wavelets which the wind of hysterical protest will one day swell to raging billows. He is certainly paving the way for socialistic demagoguery—unless, perchance, he is saving the country by preventive inoculations.

In conclusion, let me remark that social hysteria, like material disease, attacks both small and large "individual groups," or may became general; in "shop" parlance it may be epidemic, epidemic or pandemic. Its epidemic form is well illustrated by the recent panic.

Remedy? There is none. Social hysteria is rooted too far down in the depths of human nature and too thoroughly mingled with the protean morphology of psychology and human degeneracy. History has ever repeated and always will repeat itself. And, morever, in the long run, the race has taken pretty good care of itself. In all phases of social change, turmoil and progress, the remorseless law of evolution is as potent as in biology. Human history has been a procession of births, rises and falls of social systems.

